# Facial Apocrine Chromhidrosis: A Case Report

**DOI:** 10.7759/cureus.53401

**Published:** 2024-02-01

**Authors:** Ali Alhudaif, Mohammad K Almazied, Mohammed Khashoggi, Eman Alasgah, Abdullah Alhaddab

**Affiliations:** 1 Dermatology, Diriyah Hospital, Riyadh, SAU

**Keywords:** sweating, aluminum chloride, botulinum toxin a, facial, apocrine chromhidrosis

## Abstract

Apocrine chromhidrosis is a rare disease that is characterized by colored sweating. Here, we present a rare case that was successfully treated for this condition. A 32-year-old woman presented with dark blue discharge from her cheeks. She was diagnosed with apocrine chromhidrosis and was treated successfully with botulinum toxin type A.

## Introduction

Apocrine chromhidrosis is a rare disease characterized by the production of colored sweat, commonly dark blue. It is an idiopathic disease that affects apocrine glands in the face, underarms, and genital area. Chromhidrosis can be divided into apocrine, eccrine, and pseudo subtypes. It is important to rule out pseudochromhidrosis, which is caused by external factors such as dyes or bacteria; so, detailed medical history is crucial to differentiate. Chromhidrosis can affect patients psychosocially, leading to embarrassment and anxiety. It is diagnosed clinically and confirmed by skin biopsy. The treatment modalities include topicals such as aluminum chloride lotion and capsaicin cream or interventional botulinum toxin injections. [1] Here, we present a rare case of this condition with a successful treatment. A 32-year-old woman who presented with dark blue discharge from her cheeks was diagnosed with apocrine chromhidrosis and treated successfully with botulinum toxin type A.

## Case presentation

**Figure 1:**
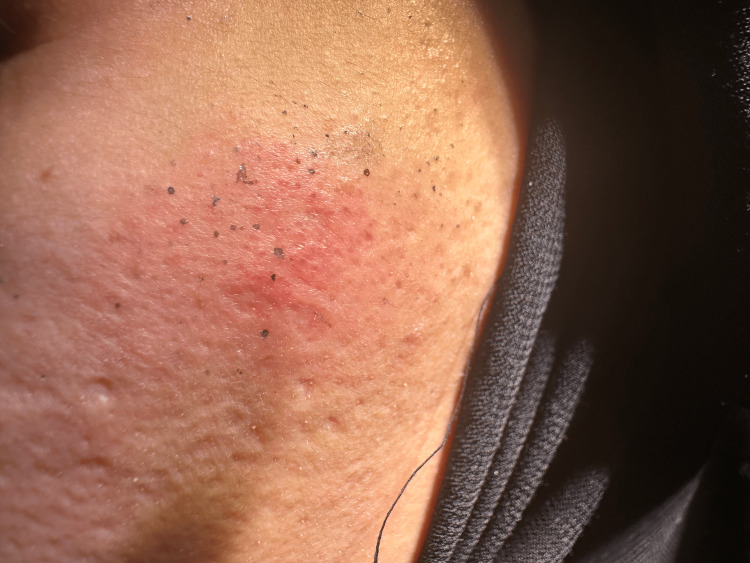
Multiple dark blue discharges over the left cheek, which is a typical presentation of chromhidrosis

A 32-year-old female, medically free, presented to the clinic complaining of dark blue discharge from her cheeks as shown in Figure 1 for one year. She had no recent history of using any medications. During a physical examination, there was a dark blue discharge from both cheeks. She was treated with topical tretinoin 0.1% every other day at night for three months with no improvement. The patient refused a skin biopsy for cosmetic reasons since it would lead to a scar on her face. The patient was treated with topical aluminum chloride 20% solution daily at night for one month with no improvement. Eventually, we treated the patient with 100 units of botulinum toxin type A mixed with 2.5 cc of normal saline; the concentration was four units per 0.1 cc. The patient was seen after three months in a follow-up clinic with no relapse. The botulinum toxin effect will decrease with time. In case of recurrence of symptoms, a second session has to be performed.

## Discussion

In this study, we report a rare finding of facial apocrine chromhidrosis in an adult woman. In this case, the characteristic features included dark blue discharge from the patient's cheeks bilaterally and therapeutic response to botulinum toxin type A. The pathology was not confirmed via skin biopsy owing to the patient's cosmetic concerns. In this study, no established etiological factors contributed to the development of facial apocrine chromhidrosis in the patient. Contrary to eccrine chromhidrosis, apocrine chromhidrosis is limited to body areas containing apocrine glands, including the areola, axilla, anogenital area, ears, scalp, eyelids, and trunk. While eccrine chromhidrosis is regarded as an exogenous process, apocrine chromhidrosis is an intrinsic phenomenon [2-4].

The colored sweat in the apocrine chromhidrosis is released in response to emotional stimuli, hot baths, and skin rubbing. The pathogenesis may also involve the substance P (SP), a neuropeptide. Compared to normal sweat in healthy individuals, patients with apocrine chromhidrosis have either a greater amount of lipofuscin or a greater extent of lipofuscin oxidation. The latter is proportional to the darkness of lipofuscin, imparting a black, brown, yellow, green, or blue color to the sweat [4,5].

Various studies in the medical literature have reported apocrine chromhidrosis in patients. Beer et al. reported a case of axillary chromhidrosis in a woman who had been suffering from the ailment for several years, and the patient was treated with botulinum toxin type A, which substantially improved her condition [6]. This is similar to our case study, where the patient was administered botulinum toxin type A and topical aluminum chloride. Wyrick et al. reported a rare finding of atypical chromhidrosis in a 44-year-old woman with a chief complaint of orange staining of clothes and bronzing of the skin, indicative of orange sweat. Furthermore, rare gram-positive cocci were also observed in the eccrine glands of the patient. Instead of botulinum toxin type A, this patient was treated with amoxicillin/clavulanate potassium 875 mg tablets with no improvement [7].

The mechanism by which botulinum toxin type A suppresses apocrine chromhidrosis is not yet established. Apocrine glands are considered to have a lower density of cholinergic innervation. The toxin can block nerve transmission, suppressing apocrine glands' stimulation. The toxin may also inhibit the release of SP [8]. Besides botulinum toxin type A, chromhidrosis can also be treated using topical capsaicin. A case study has indicated the efficacy of topical capsaicin in suppressing chromhidrosis; however, the disease returned within two days of discontinuation of this medication [9].

## Conclusions

In conclusion, we reported an unusual case of facial apocrine chromhidrosis, clinically represented by dark blue sweat over both cheeks. The patient did not improve on topical aluminum chloride. However, the patient demonstrated clinical improvement with botulinum toxin type A treatment, indicating the clinical efficacy of the latter in treating this pathology. Further treatment options include the application of topical capsaicin.
